# Retraction: Individualized Homeopathic Treatment and Fluoxetine for Moderate to Severe Depression in Peri- and Postmenopausal Women (HOMDEP-MENOP Study): A Randomized, Double-Dummy, Double-Blind, Placebo-Controlled Trial

**DOI:** 10.1371/journal.pone.0232415

**Published:** 2020-04-23

**Authors:** The PLOS ONE Editors

Following the publication and correction of this article [1, 2], questions were raised about the study design and reporting that prompted a full reassessment of the article. This post-publication evaluation involved three senior staff editors, a statistical reviewer, and a member of *PLOS ONE*’s Editorial Board, and identified the following concerns:

- The individualized homeopathic treatment (IHT) and the decision-making framework for treatment adjustments were not reported in sufficient detail to enable reproducibility of the study or an assessment as to the clinical validity of treatment choices. In response to this issue, the authors provided information in post-publication discussions about the individualized homeopathic consultations and guidance used for homeopathic prescription, and commented that potencies of homeopathic remedies were selected on the basis of homeopathic principles. Citing a 2014 systematic review of clinical trials involving IHT [3], the authors commented that the replicability of IHT has been demonstrated by previous studies that applied the same treatment approach to other medical conditions. They also asserted that the information reported in [1] was sufficient to enable replication by trained IHT practitioners, and that the study was reported per CONSORT criteria and the RedHot supplement to CONSORT for homeopathy clinical trials.
- The study design did not include sufficient controls to rule out the possibility that observed effects in the IHT group—which received homeopathic remedies at 30C (10^−60^) and 200C (10^−400^) dilutions—represent placebo effects rather than responses to the homeopathic treatments. The authors disagreed with this point, provided additional information about the methodology, and asserted that elements of the study design accounted for placebo effects.
- Insufficient information was provided in the article about the methodology by which diagnoses were made in the study, the reliability of that methodology, and how blinding was achieved during the diagnostic assessments. The article reported that participants were diagnosed according to DSM-IV but did not include details as to how the criteria were applied in practice during the trial. The authors provided additional methodological information in post-publication discussions.

The *PLOS ONE* Editors and the consulted Academic Editor reviewed the information and additional methodological details provided by authors in response to each of these issues. The Editors determined that the additional information provided did not fully address the above concerns, which call into question the reproducibility of the study, the scientific validity and rigor of the study design, and whether conclusions are adequately supported by the results. For these reasons, the *PLOS ONE* Editors retract this article. We regret that these issues were not identified and addressed prior to the article’s publication.

During the post-publication assessment, questions were also raised about whether the 6-week duration of the study and fixed dosing regimen for fluoxetine treatment were sufficient to allow an assessment as to the efficacy of fluoxetine where it was prescribed. The consulted Academic Editor noted that other randomized clinical trials using fluoxetine allow for increasing dosage in case of nonresponse. The Academic Editor also noted that per clinical practice guidelines, trials of antidepressants should generally last 8–12 weeks [4] and it generally requires 4–8 weeks of treatment to determine whether a patient is responsive to a particular intervention [5]. The authors noted that trials identified in published systematic reviews [6, 7, 8] used the same duration of fluoxetine treatment as was applied in [1]. The authors further commented that the fluoxetine dose used aligns with starting doses applied in clinical practice [4].

EdCM-C, LL-G, LA-F, and JA-B did not agree with retraction.
